# Editorial: The Legacy of Dr. Leonard D. Kohn to Thyroid Pathophysiology

**DOI:** 10.3389/fendo.2022.906340

**Published:** 2022-06-08

**Authors:** Cesidio Giuliani, Hiroki Shimura, Jae Hoon Chung, Giorgio Napolitano

**Affiliations:** ^1^ Unit of Endocrinology, Department of Medicine and Sciences of Aging, University “G. d’Annunzio” of Chieti-Pescara, Chieti, Italy; ^2^ Centre for Advanced Studies and Technology (CAST), University “G. d’Annunzio” of Chieti-Pescara, Chieti, Italy; ^3^ Department of Laboratory Medicine, School of Medicine, Fukushima Medical University, Fukushima, Japan; ^4^ Division of Endocrinology and Metabolism, Department of Medicine and Thyroid Center, Samsung Medical Center, Sungkyunkwan University School of Medicine, Seoul, South Korea

**Keywords:** Leonard D Kohn, thyroid cancer, thyroid autoimmunity, FRTL-5 cells, thyroid biology

This Research Topic honors the memory of Dr. Leonard D. Kohn (Len) (1935-2012), a prominent scientist particularly in the field of thyroid biology and thyroid autoimmunity. Len was Section Chief in the Laboratory of Biochemistry and Metabolism at the National Institute of Diabetes and Digestive and Kidney Diseases (NIDDK) of the National Institutes of Health (NIH) in Bethesda MD, USA for 25 years. He then moved to Ohio University as a Distinguished Senior Research Scientist at the Edison Biotechnology Institute. Several generations of young investigators from all over the world were trained in his laboratories and Len had an enormous impact on his trainees’ research career. The present topic collects a series of 10 articles (8 original research and 2 updated reviews) authored by Len’s former trainees. These articles cover various topics of thyroid pathophysiology and some of them (Chen et al.; Giuliani et al; Napolitano et al.) constitute the development of research projects initiated in Len’s laboratories several years ago.

In this Research Topic 4 articles (Chen et al.; Giuliani et al; Napolitano et al.; Bucci et al.) deal with thyroid autoimmunity.

The study of Chen et al. used the thyroid cell line FRTL-5 as a model to investigate the effects of Prunella Vulgaris (PV), a plant used in Chinese medicine, on innate immunity. The study shows that PV inhibits the activation of the innate immune response induced in the FRTL-5 cells by the transfection of ds-DNA and ds-RNA. It should be emphasized that the ability of ds-polynucleotides to activate, in thyroid cells, genes and pathways involved in the immune response was observed by Suzuki in Len’s laboratory at NIH (1). In their work Chen et al. show that PV treatment causes a decreased activation of nuclear factor-kB (NF-kB) and Interferon regulatory factor 3 (IRF3) and abolishes the expression of several genes involved in the immune response including the Major Histocompatibility Complex (MHC) class I.

Giuliani et al. show, using FRTL-5 cells, that the “tissue-specific” region of the MHC class I promoter has a dominant role in the regulation of the gene expression and that different hormones and factors act on it by modifying the binding of two distinct members of the transcription factors families: activator protein-1 (AP-1) and NF-kB, c-jun and p65 respectively. The study is the continuation of a research project, started 30 years ago in Len’s laboratory (2, 3), aimed at evaluating the role of MHC class I expression in thyroid diseases.

Napolitano et al. review the role of the TSH receptor antibodies (TSHrAb) in chronic autoimmune thyroiditis by analyzing the conditions under which the biological assay for TSHrAb detection can be clinically useful. It should be noted that Len’s laboratories played a pivotal role in the development of bioassays for TSHrAb (4).

Bucci et al. performed an updated review on the relationship between thyroid autoimmunity (TAI) and female infertility. In particular, the authors evaluate the role of euthyroid TAI in infertility and pregnancy outcome, also considering the role of TAI in assisted reproductive technology. This is a very interesting and controversial issue, and the authors provide a timely review of the literature.

Two articles (Ulianich et al. and Lee et al.) mainly deal with aspects of thyroid biology.

In their article Ulianich et al. show the effects of endoplasmic reticulum (ER) stress in the thyroid cell lines PCCl3 and FRT. In detail, the induction of ER stress by thapsigargin and tunicamycin results in cells dedifferentiation, loss of epithelial organization, shift towards a mesenchymal phenotype, and activation of the antioxidant response. These data show a new molecular mechanism of cell response following ER stress that can lead to a loss of thyroid function.

Lee et al. investigate the role of the primary cilium of thyrocytes in thyroglobulin (Tg) endocytosis using *“in vivo”* murine models. They demonstrated that the Lrp2/megalin complex, involved in Tg uptake, is localized in the primary cilium of thyroid follicular cells. It is interesting to note that the interaction between Tg and thyroid cell membrane was an old interest of Len (5), this interest was subsequently directed to the role of Tg in the regulation of thyroid growth and function (6, 7).

Three original research articles, from Korean researchers, deal with some clinical features of thyroid cancer.

The study by Park et al. evaluate the incidence of childhood thyroid cancer in Korea between 1999 and 2017. They found that, unlike adults, the incidence of thyroid cancer in children continues to increase. The authors also collected epidemiological data on radiation exposure, iodine intake, prevalence of obesity, and behavior habits that provide some explanation for this finding.

Park et al. conducted a retrospective study in patients with medullary thyroid carcinoma to determine the role of preoperative serum calcitonin levels in the prognosis of this disease. They define a preoperative serum calcitonin cut-off value to predict structural recurrence. This cut-off value is also associated with the clinical outcome.

In their work Lee et al. show the adverse effect of TSH-suppressive therapy in patients over 60 years old thyroidectomized for differentiated thyroid cancer. They found that low serum TSH concentrations are associated with a lower grip strength particularly in patients between the age of 60 and 70. These findings are important given the association between low grip strength and cardiovascular morbidity, as well as all-cause mortality (8).

Finally, a clinical study by Sohn et al. focuses on hypothyroidism. The authors address all-cause mortality in hypothyroid patients undergoing treatment with levothyroxine. They report that the mortality rate is higher in the hypothyroid patients compared to the control, with the highest risk within 1 year of treatment. Possible explanations of these results are discussed.

In conclusion, this Research Topic constitutes a tribute to the memory of Len by some of his former research fellows from the Countries that were most represented in Len’s laboratories: Japan, Italy and Korea. The Research Topic collects both original research and updated reviews on thyroid biology and clinic. The variety of the issues covered in this Research Topic reflects Len’s scientific broad-mindedness.

## Author Contributions

All authors listed have made a substantial, direct, and intellectual contribution to the work and approved it for publication.

## Conflict of Interest

The authors declare that the research was conducted in the absence of any commercial or financial relationships that could be construed as a potential conflict of interest.

## Publisher’s Note

All claims expressed in this article are solely those of the authors and do not necessarily represent those of their affiliated organizations, or those of the publisher, the editors and the reviewers. Any product that may be evaluated in this article, or claim that may be made by its manufacturer, is not guaranteed or endorsed by the publisher.
